# Identification and functional analysis of N6‐methyladenine (m^6^A)‐related lncRNA across 33 cancer types

**DOI:** 10.1002/cam4.5001

**Published:** 2022-07-04

**Authors:** Dahua Xu, Zhizhou Xu, Xiaoman Bi, Jiale Cai, Meng Cao, Dehua Zheng, Liyang Chen, Peihu Li, Hong Wang, Deng Wu, Jun Yang, Kongning Li

**Affiliations:** ^1^ Key Laboratory of Tropical Translational Medicine of Ministry of Education College of Biomedical Information and Engineering, Hainan General Hospital, Hainan Medical University Haikou China; ^2^ School of Life Sciences, Faculty of Science The Chinese University of Hong Kong Hong Kong

**Keywords:** ceRNA, drug sensitivity, lncRNA, m^6^A, pan‐cancer

## Abstract

**Background:**

N6‐methyladenosine (m^6^A) plays an essential role in tumorigenesis and cancer progression. Long noncoding RNAs (lncRNAs) are discovered to be important targets of m^6^A modification, and they play fundamental roles in diverse biological processes. However, there is still a lack of knowledge with regards to the association between m^6^A and lncRNAs in human tumors.

**Methods:**

The relationship between lncRNAs and 21 m^6^A regulators was comprehensively explored, through the integration of multi‐omics data from M6A2Target, m6A‐Atlas, and TCGA (The Cancer Genome Atlas). In order to explore the potential roles of m6A‐related lncRNAs in human tumors, three applicable methods were introduced, which include the construction of ceRNA networks, drug sensitivity estimation, and survival analysis.

**Results:**

A substantial number of positive correlation events across 33 cancer types were found. Moreover, cancer‐specific lncRNAs were associated with tissue specificity, and cancer‐common lncRNAs were conserved in cancer‐related biological function. In particular, the m^6^A‐related lncRNA FGD5‐AS1 was found to be associated with cancer treatment, through its influence on cisplatin resistance in breast cancer patients. Finally, a user‐friendly interface Lnc2m6A, which is enriched with various browsing sections resource for the exhibition of relationships and putative biogenesis between lncRNAs and m^6^A modifications, is offered in http://hainmu‐biobigdata.com/Lnc2m6A.

**Conclusions:**

In summary, the results from this paper will provide a valuable resource that guides both mechanistic and therapeutic roles of m^6^A‐related lncRNAs in human tumors.

## Graphical abstract

We comprehensively explored the relationships between lncRNAs and m6A regulators across 33 cancer types. The results from this paper will provide a valuable resource that guides both mechanistic and therapeutic roles of m6A‐related lncRNAs in human tumors.

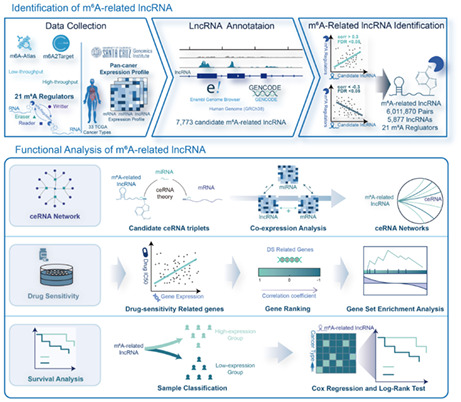

## 1 INTRODUCTION

As the most abundant modification for RNA molecules, N6‐methyladenosine (m^6^A) plays an essential role in RNA stability, translation, and even the carcinogenic process.^1^, ^2^ The rounded system of m^6^A is determined by multiple m^6^A regulators comprised of “writers” (methyltransferases), “readers” (m^6^A‐bingding proteins), and “erasers” (demethylases).^3^ Exploration of the genetic alteration and expression dysfunction for m^6^A regulators and its related molecules has successfully detected RNA methylation‐based therapeutic targets.^4^ In view of the involvement in important biological processes, Li et al. systematically analyzed the molecular characterization and clinical relevance of m^6^A regulators across human cancers.^5^ However, a comprehensive landscape and functional analysis of long noncoding RNA (lncRNA), a crucial RNA molecule in which m^6^A is highly modified, with m^6^A regulators in the neoplastic field is still lacking.

Dysregulation of lncRNA is associated with the pathogenicity and progress of malignancy.^6^, ^7^ A growing number of researchers have demonstrated that m^6^A is a critical internal modification, which is implicated in the dysfunction of lncRNA. For example, the upregulation of lncRNA LCAT3 is mediated by the m^6^A writer METTL3, thus recruiting FUBP1 to activate c‐MYC and promote proliferation and invasion in lung cancer cells.^8^ METTL3 could also increase the expression of lncRNA PCAT6 in an IGF2BP2‐dependent manner, it then enhances the stability of IGF1R mRNA and promotes bone metastasis in prostate cancer.^9^ Through bioinformatics analysis, Tu et al. systematically identified m^6^A‐related lncRNA with prognosis capacity for lower‐grade glioma, by the employment of the TCGA and CGGA data sets.^10^ Therefore, integrating associations of lncRNA and m^6^A regulators will provide newly methylated biomarkers and help understand the potential coregulate mechanism.

It has been recognized that m^6^A could participate in the lncRNA‐mediated competing endogenous RNA (ceRNA). A model where lncRNA competed with other RNAs to bind to miRNA, thereby influencing the biological processes in the tumor. Zheng et al. have revealed the upregulated lncRNA FAM225A mediated by m^6^A modifications, could promote cell proliferation, invasion, and migration, by sponging miR‐590‐3p and miR‐1275 in nasopharyngeal carcinoma.^11^ In another case, the lncRNA LINC00958 could be positively regulated by METTL3, and it promoted lipogenesis as well as the progression through the competing miR‐3619‐5p/HDGF axis in hepatocellular carcinoma.^12^ Meanwhile, the alterations of the ceRNA relationship strongly affect drug sensitivity and, in many cases, are potential biomarkers for response to drugs. For instance, Liu et al. systematically constructed drug resistance‐related ceRNA interactions of lncRNA and mRNA across 19 cancer types and revealed the potential mechanism of lncRNA GAS5 and RPL8 in drug resistance.^13^ Moreover, the dysregulated lncRNA‐mediated ceRNA triple MEG3/hsa‐miR‐200b‐3p/AKT2 was associated with the sensitivity for doxorubicin in osteosarcoma.^14^ Therefore, these clues offer an opportunity to explore the potential mechanism among m^6^A modifications, lncRNA‐mediated ceRNA, and drug sensitivity.

In this study, a systematic evaluation of the lncRNAs and m^6^A regulators was performed, by taking the advantage of published m^6^A‐related resources and omics data of 33 cancer types from the TCGA cohort. The landscape of m^6^A‐related lncRNAs was delineated, and the differences between lncRNA categories in multiple genomic features were revealed. Cancer‐specific lncRNAs which were associated with tissue specificity were found, and cancer‐common lncRNAs were conserved in cancer‐related biological function. Moreover, key essential lncRNA FGD5‐AS1 which was strongly related to m^6^A regulators served as a novel biomarker for drug targets. Finally, a valuable resource for identifying and investigating the function of m^6^A‐related lncRNAs in cancer was built. The findings will promote the understanding of the regulatory mechanism between m^6^A and lncRNA and further help researchers identify noteworthy epigenetic biomarkers for tumor therapy.

## 2 METHODS

### 2.1 Data collection and preprocessing

Twenty‐one m^6^A regulators from previous studies were collected, which includes 11 readers (HNRNPA2B1, HNRNPC, IGF2BP1, IGF2BP2, IGF2BP3, RBMX, YTHDC1, YTHDC2, YTHDF1, YTHDF2, and YTHDF3), eight writers (METTL14, METTL16, METTL3, RBM15, RBM15B, VIRMA, WTAP, and ZC3H13), and two erasers (ALKBH5 and FTO). The transcriptome profiles and clinical information for more than 10,000 patients, across 33 cancer types by TCGA Pan‐Cancer (PANCAN) cohort, were downloaded from UCSC Xena (http://xena.ucsc.edu/). Genome‐wide annotations of lncRNAs and mRNAs were obtained from GENCODE (V35, GRCh38). Only expressed genes with TPM > 0 in at least 70% of samples were retained. All of the expression profiles were log2 transformed. The detailed information and sample numbers for the PANCAN cohort are shown in Table S1.

The experimental human miRNA–lncRNA/mRNA interactions were downloaded from miRTarBase 2018,^15^ TarBase V7,^16^ miRecords V4,^17^ lncRNASNP2,^18^ StarBaseV2.0,^19^ and LncBaseV2.^20^ After standardization and redundancy analysis, 920,789 miRNA–mRNA pairs and 28,395 miRNA–lncRNA pairs were obtained, which included 1395 miRNAs, 2382 lncRNAs, and 18,247 mRNAs.

The drug response data were collected from Genomics of Drug Sensitivity in Cancer (GDSC, https://www.cancerrxgene.org/). This includes the half‐maximal inhibitory concentration (IC50) values and transcriptome profiles for 988 cell lines corresponding to 18 TCGA cancer types and 450 drugs.

External gene expression profiles for 11 cancers of 7306 samples were collected from the merged microarray‐acquired data sets (MMDs, Affymetrix Human Genome U133 Plus 2.0), which were processed uniformly through RMA normalization and batch effect corrected.^21^ The peaks of m^6^A/MeRIP‐seq located in lncRNAs for multiple cancer cell lines were obtained from REPIC (https://repicmod.uchicago.edu/repic/index.php).^22^

### 2.2 Identification of m^6^A‐related lncRNAs

To identify the associations between m^6^A regulators and lncRNAs in human tumors, the well‐known m^6^A‐related databases M6A2Target and m6A‐Atlas were integrated. This provides high‐confidence targets for m^6^A regulators.^23^, ^24^ Based on the potential 163,233 m^6^A–lncRNA pairs, Pearson correlation analysis was applied to mine the m^6^A‐related lncRNAs in specific cancer types. LncRNAs with absolute correlation coefficient *r* > 0.3 and normalized *p* < 0.05, after false discovery rate (FDR) correction, were considered m^6^A‐related lncRNAs.

### 2.3 Genomic features of lncRNAs

The m^6^A DRACH motifs for lncRNAs were predicted by SRAMP.^25^ The evolutionary conservation scores for lncRNAs were obtained through R package phastCons100way.UCSC.hg38 (3.7.1).^26^ The normalized CpG fraction (observed CpG/expected CpG) was calculated based on the sequences of lncRNAs, where the expected CpG was calculated as (GC content/2)^2^.^27^ Differences in conservation scores, the number of CpGs, and motifs between different types of lncRNAs were evaluated with Student's *t* test. The genomic differences for normalized CpG fractions were evaluated by Kolmogorov–Smirnov tests.

### 2.4 Construction of m^6^A‐mediated ceRNA networks

The candidate lncRNA‐miRNA‐mRNA axis was built, based on the experimental miRNA regulations. Next, the m^6^A‐related lncRNAs in an independent cancer type were selected to construct the m^6^A‐mediated ceRNA networks. Only the triple with positive correlation between m^6^A‐related lncRNAs (*r* > 0.3 and FDR < 0.05) and mRNAs and negative correlation between miRNAs and lncRNAs/mRNAs (*r* < −0.3 and FDR < 0.05) were identified as ceRNA interactions. To further explore the potential biological function of m^6^A‐related lncRNAs, the mRNAs involved in m^6^A‐mediated ceRNA networks were passed to Metascape (http://metascape.org/) with the setting of species (“Homo sapiens”).^28^

### 2.5 Estimation of the relationship between m^6^A‐related lncRNAs and drug resistance

To explore the potential role of m^6^A‐related lncRNAs in drug resistance, a computational method was proposed, which integrates m^6^A‐mediated ceRNA interactions and drug response data. First, all mRNAs were ranked based on the *r* between their expression and drug IC50 in an independent cancer type. Then, the ranked gene list $L=\left\{g_{1},g_{2},g_{3},\ldots ,g_{N}\right\}$ was subjected to each lncRNA‐involved ceRNA network and calculated the enrichment score (ES) based on GSEA theory.^29^ The content percentage of genes present was evaluated with genes in ceRNA network *S* (“hits”) weighted against genes, not in *S* (“misses”), up to a given position *i* in *L* according to the following formula:

$$P_{\mathrm{hit}}\left(S,i\right)=\sum_{\begin{array}{c} g_{j}\in S\\ j\leq i \end{array}}\frac{{\left|r_{j}\right|}^{p}}{N_{R}},\text{where}\;N_{R}=\sum_{g_{j}\in S}{\left|r_{j}\right|}^{p}$$

$$P_{\text{miss}}\left(S,i\right)=\sum_{\begin{array}{c} g_{j}\notin S\\ j\leq i \end{array}}\frac{1}{\left(N-N_{H}\right)}$$

The ES score was the maximum deviation from zero of $P_{\mathrm{hit}}-P_{\text{miss}}$. The significance of an ES score was assessed by comparing it with the random score for 1000 permutations. The lncRNA‐drug pairs with positive ES score and FDR < 0.05 were indicated as drug‐resistant. lncRNAs with negative ES score and FDR < 0.05 were considered drug‐sensitive. These processes were performed using the “GSEA” function in R package clusterProfiler V3.18.1.^30^

### 2.6 Survival analysis

The Cox regression was performed to assess the clinical association for each m^6^A‐related lncRNA. The tumor patients were divided into separate groups based on the median expression level of lncRNAs. The difference in overall survival (OS) for these groups was compared using the log‐rank test.

## 3 RESULTS

### 3.1 Scheme of identification and functional analysis of m^6^A‐related lncRNAs

**FIGURE 1:**
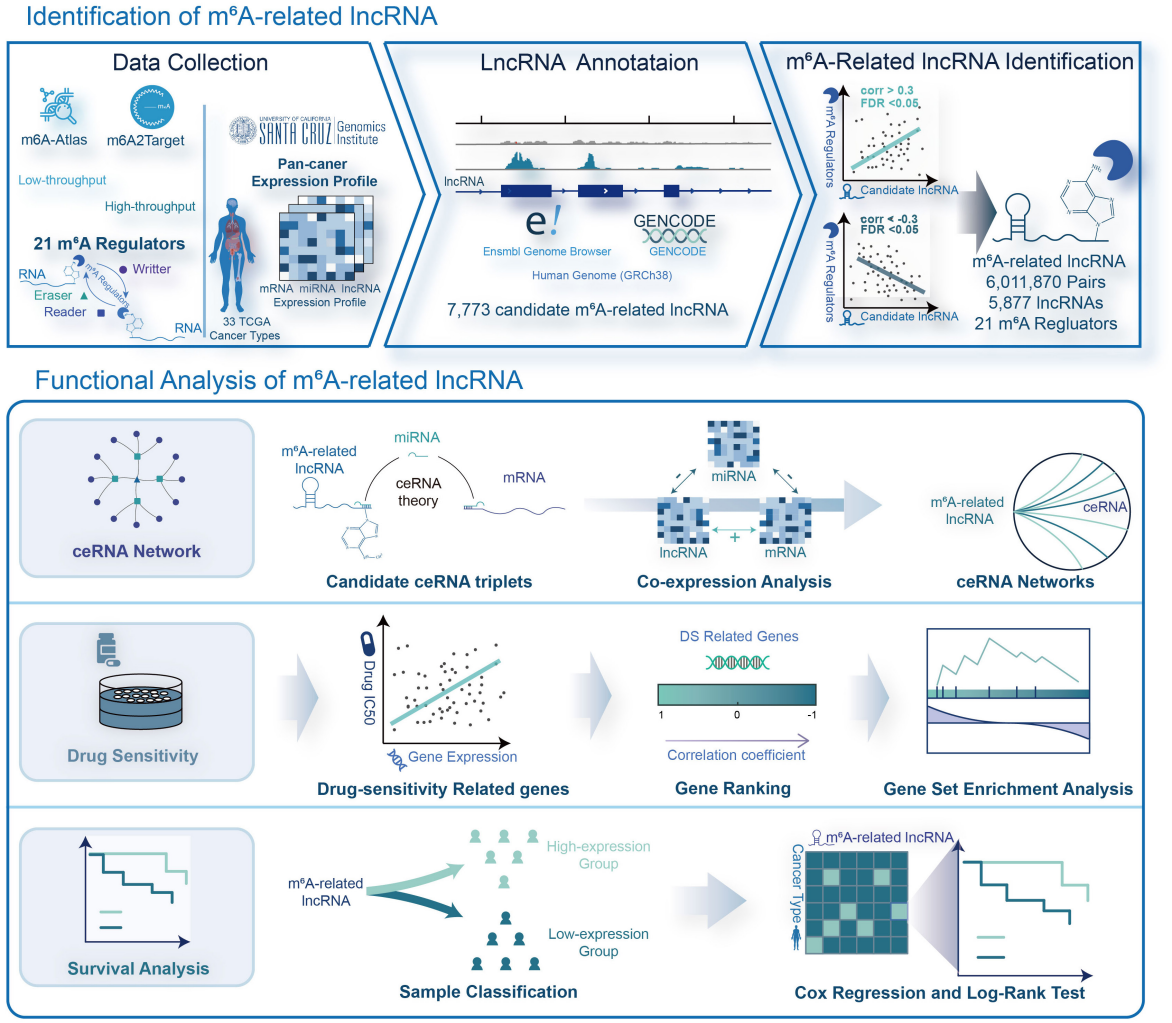
A systematical framework for the identification and functional analysis of m^6^A‐related lncRNAs. The upper panel is the workflow of identification of m^6^A across 33 cancer types. For functional analysis, we constructed ceRNA networks, estimated drug sensitivity using GSEA theory with ceRNAs, and performed survival analysis to predict the potential roles of m^6^A‐related lncRNAs.

The schematic of identification and functional analysis of m^6^A‐related lncRNAs is shown in Figure 1. The genome‐wide m^6^A‐binding sites identified from multiple technologies for Homo sapiens were first collected. Through the annotation process, 7773 lncRNAs were filtered as candidate m^6^A‐related lncRNAs. The regulatory effects of m^6^A modifications on lncRNAs are primarily determined by m^6^A regulators. This includes readers, writers, and erasers.^3^, ^31^ To identify m^6^A‐related lncRNAs in the context of human tumors, transcriptome expression profiles from TCGA were integrated, and the associations between lncRNAs and m^6^A regulators were then estimated. In total, 6,011,870 pairs including 5877 lncRNAs and 21 m^6^A regulators were significantly correlated (|Pearson *r*| > 0.3, FDR < 0.05) across 33 cancer types. Potential functional roles of these lncRNAs were further inferred, by proposing three applicable methods: (1) constructing m^6^A‐mediated ceRNA networks, (2) estimating drug sensitivity using GSEA theory with ceRNAs, and (3) comparing the survival difference between groups classified by lncRNA. In this way, 1 ~ 359, 13 ~ 163, and 59 ~ 1357 lncRNAs were related to ceRNA networks, drug sensitivity, and clinical survival across 33 cancer types separately (Figure S1 and Table S2).

### 3.2 Landscape of m^6^A‐related lncRNAs in cancer

**FIGURE 2:**
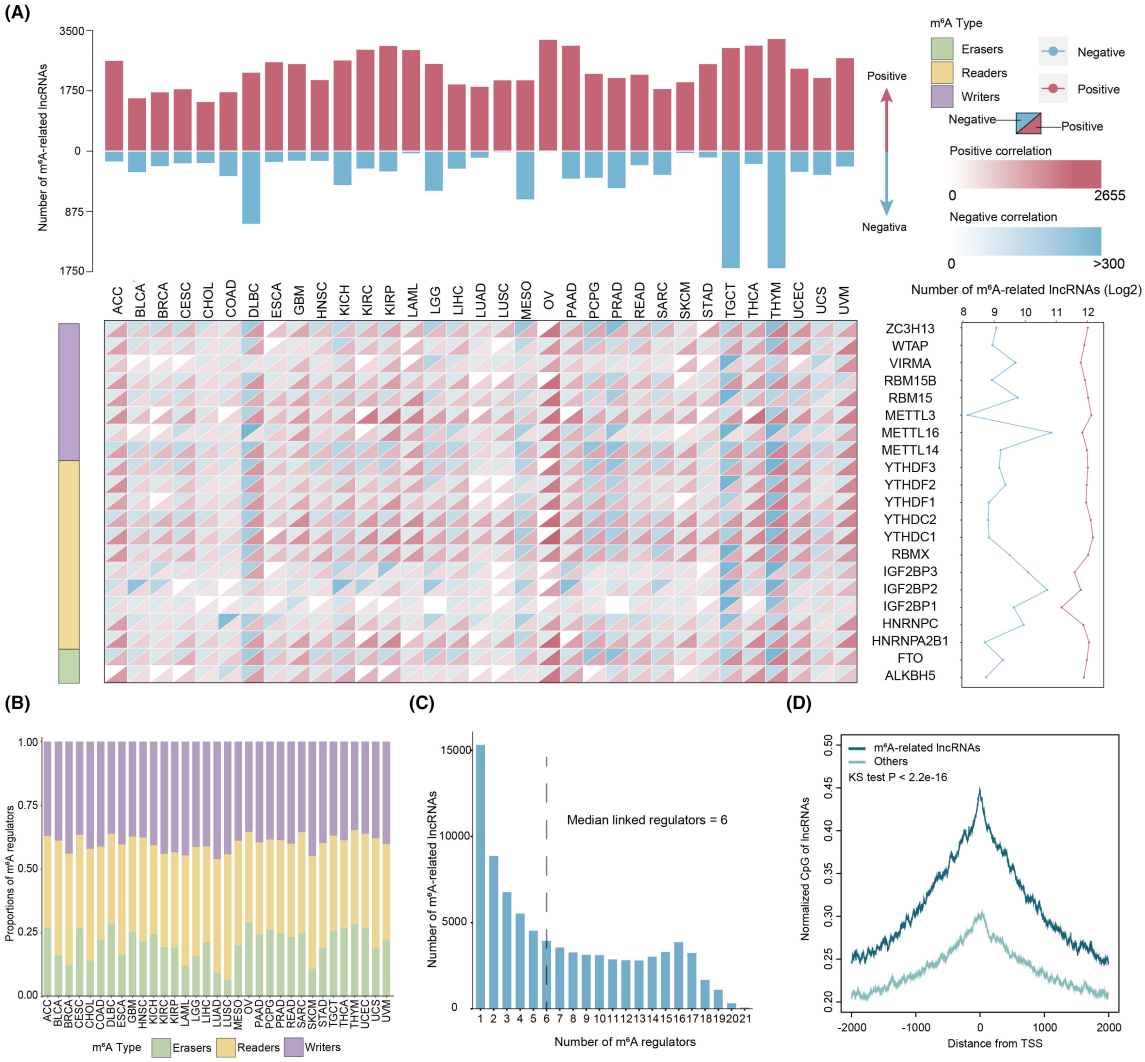
Perturbation of m^6^A‐related lncRNAs across cancer types. (A) The landscape of m^6^A‐related lncRNAs in 33 cancer types. Top: the number of positive correlation and negative correlation lncRNAs across cancer types. Bottom: the number of m^6^A‐related lncRNAs for each m^6^A regulator in 33 cancer types. Right: the total number of m^6^A‐related lncRNAs for each regulator. (B) The proportion of different regulators for regulating lncRNAs across cancers. (C) Histogram showing the number of lncRNAs that have 1 through 21 m^6^A regulators. (D) Distributions of normalized CpG around the TSSs for m^6^A‐related and other lncRNAs.

To explore the global distribution of m^6^A‐related lncRNAs across cancer types, the relationship between 21 m^6^A regulators (11 readers, 8 writers, and 2 erasers) and lncRNAs in the TCGA cohort was systematically investigated (Figure 2A). Throughout all cancer types, a substantial number of positive correlation events were found (range from 1438 to 3266), while a limited negative correlation between m^6^A regulators and lncRNAs was observed (range from 13 to 1706). There were considerable negative associations in testicular germ cell tumors (TGCT, 1704) and thymoma (THYM, 1704), while extreme few numbers in acute myeloid leukemia (LAML, 40), lung squamous cell carcinoma (LUSC, 25), ovarian serous cystadenocarcinoma (OV, 13), and skin cutaneous melanoma (SKCM, 36) (Figure 2A, top). The number of lncRNAs correlated with each m^6^A regulator was then calculated. Consistent numbers of lncRNAs were found to be positively correlated with global regulators. In contrast, higher numbers of lncRNAs were found to be negatively correlated with METTL16 and IGF2BP2, while a lower number for METTL3 was observed (Figure 2A, bottom).

The difference in the proportion of lncRNAs was then explored for three different m^6^A categories across cancer types. Although the numbers of erasers were imbalanced, there was a considerable proportion of lncRNAs across cancer types (Figure 2B). Approximately 50% lncRNAs exhibited readers‐correlated, and 40% lncRNAs exhibited writers‐correlated across cancer subtypes (Figure 2B). An abundant number of associations between lncRNA and m^6^A regulators were identified, with a median of six regulators linked to each lncRNAs (Figure 2C). These results were consistent with previous findings, in which the m^6^A regulators were inclined to interact and function with each other in a tumor context.^5^, ^32^ Since genes enriched for m^6^A modifications preferentially have CpG‐rich promoters,^33^ these genomic features were compared between m^6^A‐related and other lncRNAs. This study found that m^6^A‐related lncRNAs were significantly higher GC content and number of CpGs than the others (Figure S2A,B, *p* < 0.05). The normalized CpGs of lncRNAs were then calculated in their promoter regions, as the number of CpG might be affected by the GC content and the length of lncRNAs. The normalized CpGs were symmetric around the lncRNA promoters, and m^6^A‐related lncRNAs were also with significantly higher normalized CpGs than the others (Figure 2D, *p* < 0.05). Moreover, the numbers of DRACH motifs for the m^6^A‐related lncRNA were significantly higher than the others (Figure S2C, *p* < 0.05). It was also discovered that the m^6^A‐related lncRNAs were commonly shared between TCGA and MMDs data sets (Figure S3). In conclusion, these results suggest that there are prevalent m^6^A‐related lncRNAs across cancer types.

### 3.3 Identification and characterization of cancer‐common and cancer‐specific m^6^A‐related lncRNAs

**FIGURE 3:**
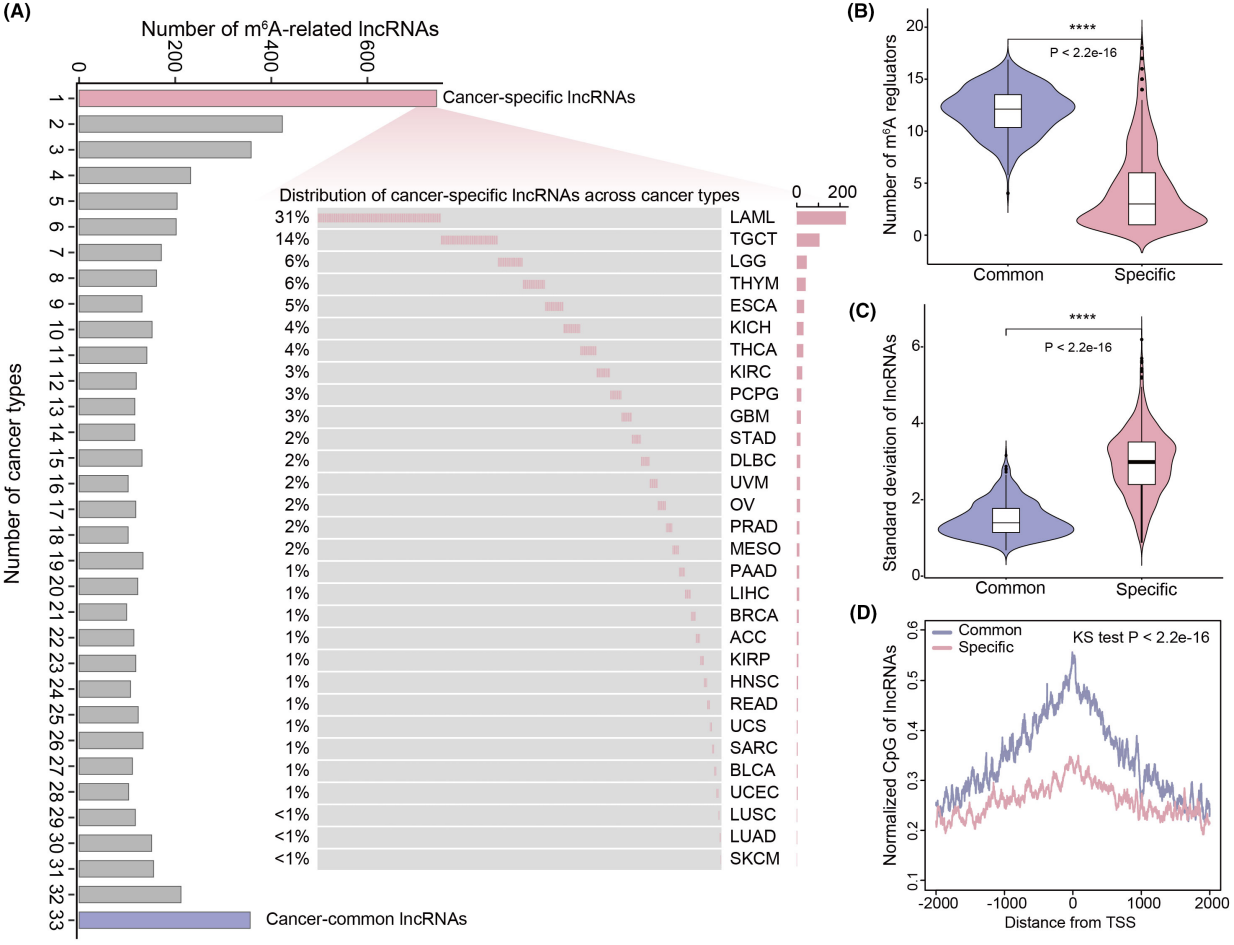
Characterization of cancer‐specific and cancer‐common lncRNAs. (A) Distribution of m^6^A‐related lncRNAs across cancer types. Red denoted m^6^A‐related lncRNAs presented in individual tumors as cancer‐specific, blue denoted m^6^A‐related lncRNAs occurred across all human tumors as cancer‐common. The waterfall plot shows the distribution of cancer‐specific lncRNAs across cancer types. (B) The comparison of the number of related m^6^A regulators between cancer‐common and cancer‐specific lncRNAs. (C) The comparison of the expression deviation between cancer‐common and cancer‐specific lncRNAs. (D) Distributions of normalized CpG around the TSSs for cancer‐common and cancer‐specific lncRNAs.

Growing evidence suggests that cancer‐common/cancer‐specific epigenetically regulated lncRNAs play essential roles in tumorigenesis and progress.^34^, ^35^ This study determined that m^6^A‐related lncRNAs occurred across all human tumors as cancer‐common lncRNAs and presented in the individual tumor as cancer‐specific. In total, 356 cancer‐common lncRNAs and 744 cancer‐specific lncRNAs were identified, which were related to m^6^A modifications in human tumors (Figure 3A). Since lncRNAs with resistant and consistently epigenetic patterns exhibited different genomic characteristics,^36^ the evolutional and genomic features of lncRNAs in cancer‐common and cancer‐specific groups were then compared. This study found that cancer‐common lncRNAs were with a significantly larger number of related m^6^A regulators and higher conservation scores than cancer‐specific lncRNAs (Figure 3B and Figure S4, *p* < 0.05). Xiao et al found that the expression of genes with m^6^A modifications occurred in more tissue types were relatively more stable, and genes with more stable expression levels were also more likely to have a higher proportion of transcripts with the m^6^A modifications.^33^ Similarly, the expression deviations of cancer‐common lncRNAs were significantly lower than cancer‐specific lncRNAs (Figure 3C, *p* < 0.05). In addition, cancer‐common lncRNAs have higher normalized CpGs in the lncRNAs promoter region (Figure 3D, *p* < 0.05).

**FIGURE 4:**
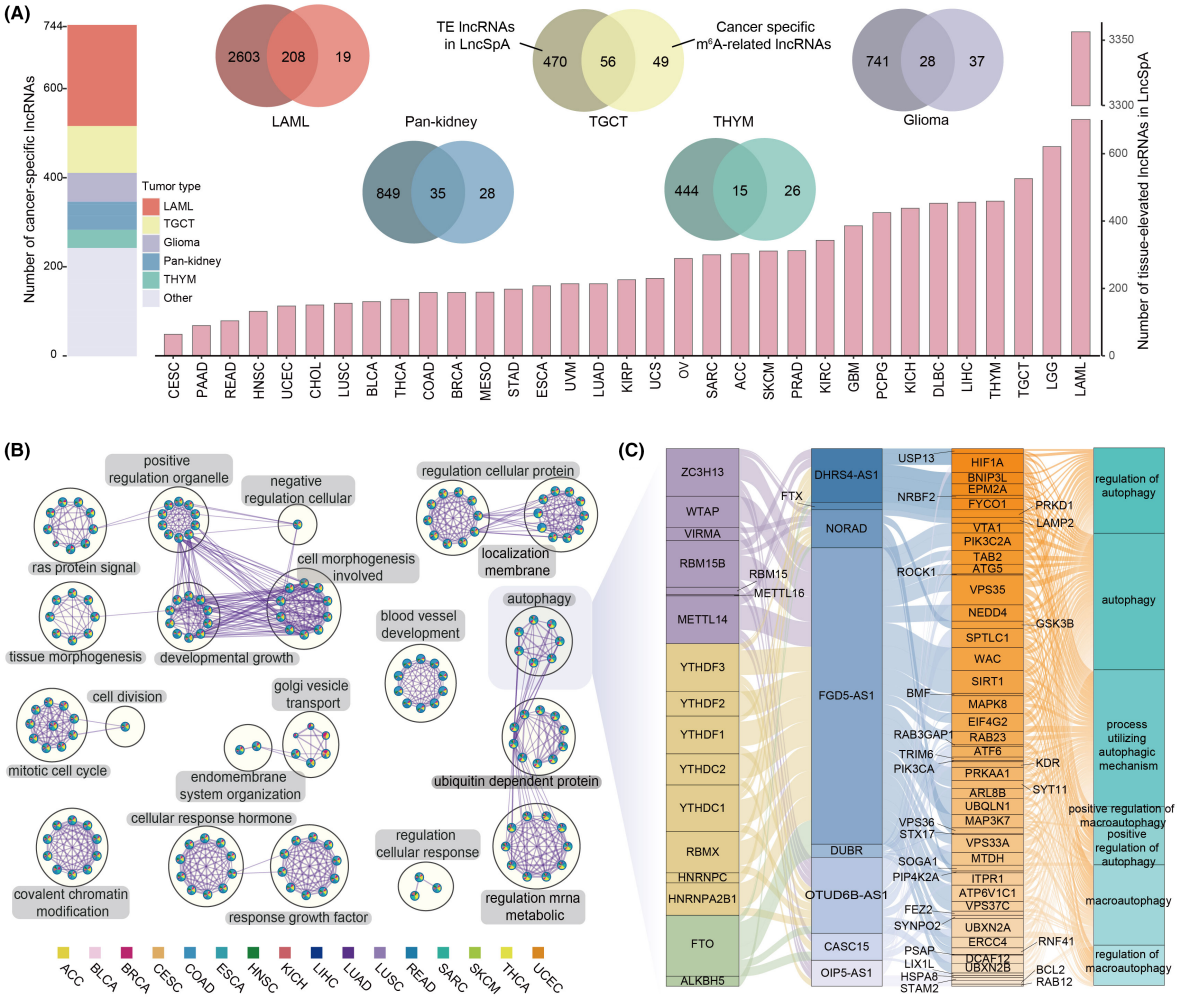
Putative regulation of cancer‐common and cancer‐specific lncRNAs biogenesis. (A) The number of cancer‐specific m^6^A‐related lncRNAs and tissue‐elevated lncRNAs across cancer types. Pie charts reflect the overlap between cancer‐specific and TE lncRNAs in LAML, TGCG, Giloma, Pan‐kidney, and THYM cancers. (B) Biological processes enriched by the essential cancer‐common lncRNAs based on ceRNA networks across cancer types. (C) The crosstalk among m^6^A regulators, lncRNAs, target genes, and autophagy pathways.

While tissue‐elevated (TE) lncRNAs were found to be associated with m^6^A modification across tissues,^37^ the relationship between the m^6^A‐related cancer‐specific lncRNAs and tissue specificity is unknown. This study found that LAML, TGCT, brain lower‐grade glioma and glioblastoma multiforme (LGG and GBM, glioma cohort), kidney chromophobe, kidney renal clear cell carcinoma, kidney renal papillary cell carcinoma (KICH, KIRC, and KIRP, Pan‐kidney cohort), and THYM possessed higher proportion cancer‐specific lncRNAs (lncRNAs number > 40) (Figure 4A). Then the LncRNA Spatial Atlas (LncSpA) was searched, and it was found that cancer‐specific lncRNAs were significantly enriched in TE lncRNAs (Figure 4A, hypergeometric test, *p* < 0.001).^38^ These observations were consistent with the results of a recent study, where it was found that brain tissues have a higher proportion of m^6^A‐modified TE lncRNAs.^37^

For cancer‐common lncRNAs, 37 cancer‐related lncRNAs were obtained from lnc2Cancer V3.0,^39^ and functional enrichment analysis was performed based on the ceRNAs in which lncRNAs linked with individual cancer types through Metascape.^28^ Although any two cancer types shared a limited proportion of ceRNAs (Figure S5), common lncRNAs were enriched in similar cancer‐related biological processes, such as RAS signal, mRNA metabolic, etc. (Figure 4B). In particular, this study found that the common lncRNAs possessed a large proportion of autophagy pathways in colorectal cancer (CRC), which includes colon adenocarcinoma (COAD) and rectum adenocarcinoma (READ) (Figure 4C). An autophagy‐mediated ceRNA–ceRNA interaction network in CRC was constructed in a previous study.^40^ These observations may provide a potential connection among m^6^A modification, autophagy, and ceRNAs.

### 3.4 Key m^6^A‐related lncRNA FGD5‐AS1 was associated with tumorigenesis and therapy

**FIGURE 5:**
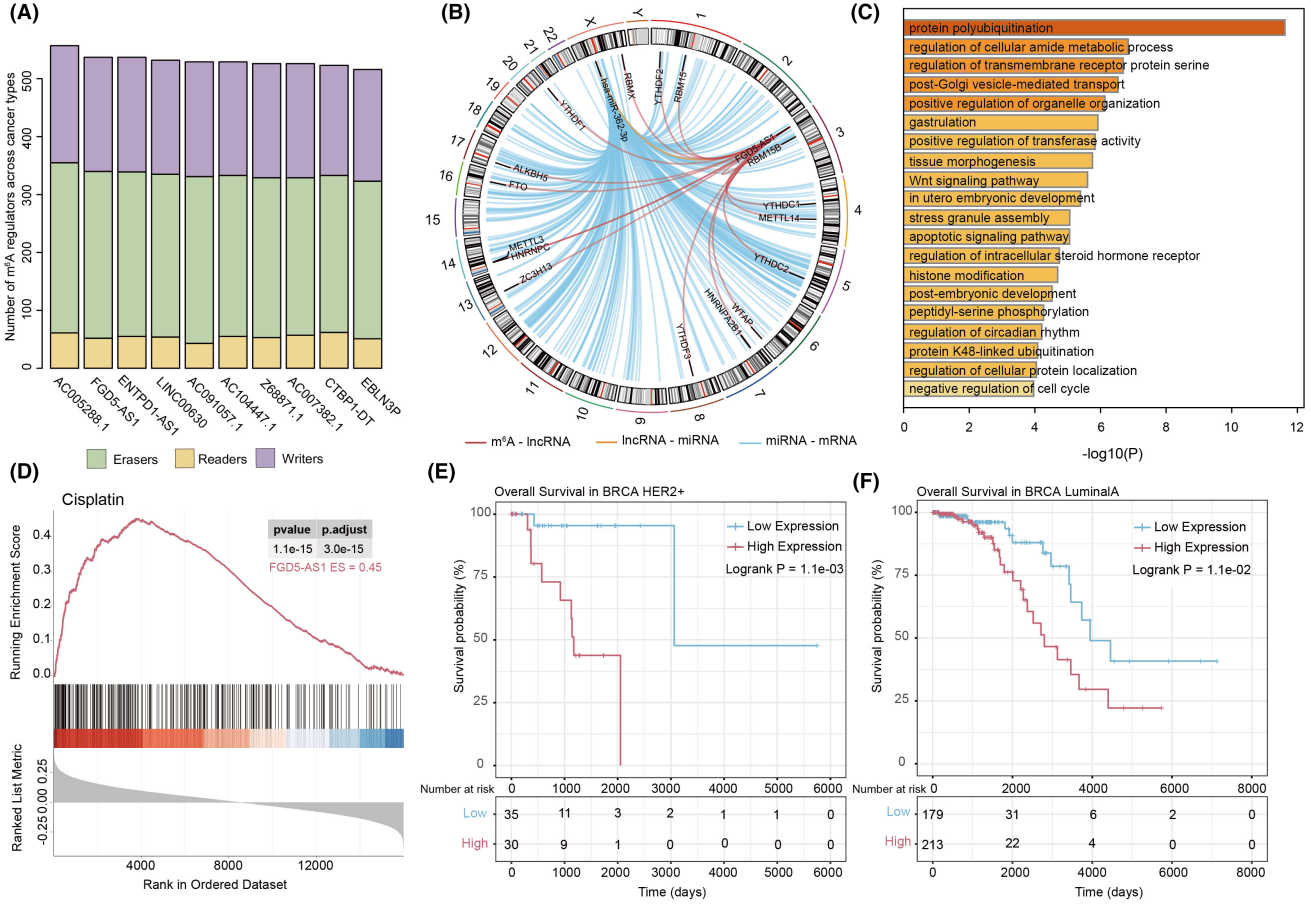
Functional characterization of FGD5‐AS1 in BRCA. (A) The top 10 m^6^A‐related lncRNAs across cancer types. (B) The layout of the chromosomal location of 21 m^6^A regulators and FGD5‐AS1‐related ceRNA network in BRCA. (C) Biological processes enriched by the FGD5‐AS1‐related ceRNA network in BRCA. (D) FGD5‐AS1 with positive enrichment in cisplatin resistance in BRCA patients. (E) and (F) Kaplan–Meier estimates of the OS based on FGD5‐AS1 expression in BRCA HER2+ and LuminalA patients.

Through analysis of all m^6^A regulators frequency across 33 cancer types in TCGA, it was found that a well‐known lncRNA, FGD5 antisense RNA 1 (FGD5‐AS1), had the second‐highest correlativity among all m^6^A regulators (Figure 5A). The expression level of FGD5‐AS1 was significantly correlated with m^6^A regulators in both TCGA and MMDs data sets (Figure S6). The expression level of FGD5‐AS1 was significantly different in lymphoid neoplasm diffuse large B‐cell lymphoma (DLBC), GBM, KIRC, LGG, pancreatic adenocarcinoma (PAAD), and THYM (Figure S7A). Moreover, the expression level of FGD5‐AS1 was associated with OS in multiple cancers. High expression of FGD5‐AS1 indicated better survival in KIRC and worse survival in liver hepatocellular carcinoma (LIHC) (Figure S7B). The functional enrichment analysis indicated that FGD5‐AS1‐related ceRNAs enriched essential biological processes such as protein polyubiquitination and regulation of cellular amide metabolic process. (Figure S7C). Moreover, numerous peaks of m^6^A/MeRIP‐seq were found to be located in the FGD5‐AS1 region for multiple cancer cell lines (Table S3). Collectively, FGD5‐AS1 played an important role in carcinogenesis and cancer progression.

FGD5‐AS1 has been found to act as an oncogene and serve a key role in glycolysis and tumor progression through the ceRNA mechanism in breast cancer.^41^ In the case of breast invasive carcinoma (BRCA, 1098 samples) in the TCGA cohort, 16 m^6^A regulators were found to be significantly associated with FGD5‐AS1, and 311 mRNAs were regulated by FGD5‐AS1 through competitively binding to has‐miR‐362‐3p (Figure 5B). The miRNA miR‐362‐3p was found to strongly influence cellular proliferation, migration, and invasion, thereby suppressing tumor growth in human breast cancer.^42^ Moreover, genes in FGD5‐AS1‐related ceRNAs were significantly enriched in protein polyubiquitination and regulation of the cellular amide metabolic process (Figure 5C). These results suggest a potential role of FGD5‐AS1 in drug resistance. Several studies have proved that FGD5‐AS1 could increase cisplatin resistance in multiple cancer types through ceRNA interactions. For instance, the FGD5‐AS1/miR‐497‐5p/SEPT2 axis could accelerate cancer progression, and increase cisplatin resistance in laryngeal squamous cell carcinoma.^43^ FGD5‐AS1 could also suppress cisplatin sensitivity of lung adenocarcinoma cells via regulating miR‐142‐5p/PD‐L1.^43^ Here, a GSEA‐based method was proposed to estimate the relationship between lncRNA and drug resistance through ceRNA networks. This study found that lncRNA FGD5‐AS1 was significantly associated with cisplatin resistance in BRCA patients (Figure 5D). In addition, the expression level of FGD5‐AS1 was strongly related to the clinical of BRCA HER2+ and LuminaA subtypes, where the lower expression of FGD5‐AS1 indicated a favorable prognosis (Figure 5E,F). In summary, the results from this study provided novel m^6^A‐related biomarkers for cancer treatment and drug development.

### 3.5 Lnc2m6A: A web‐based resource for m^6^A‐related lncRNAs in cancer

**FIGURE 6:**
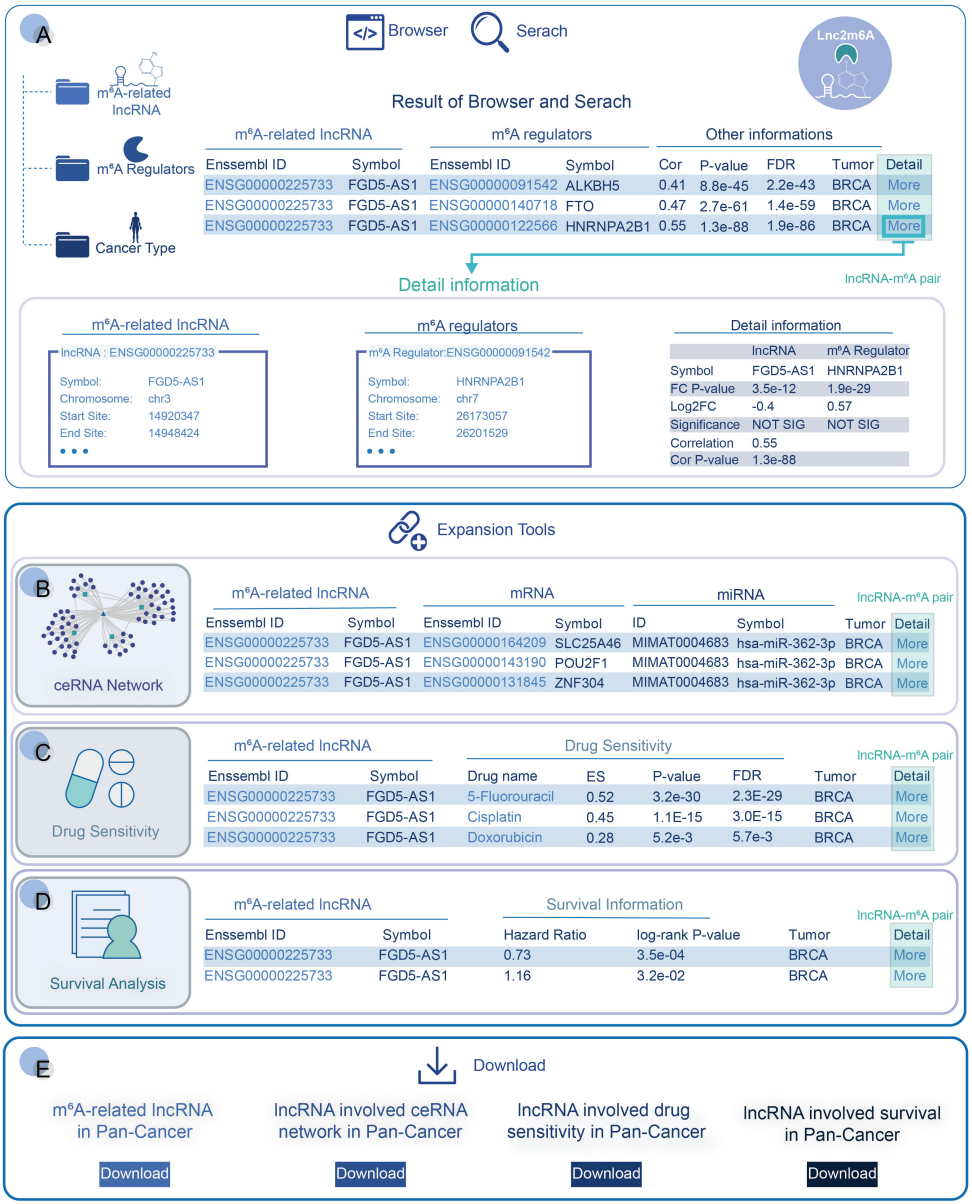
Diagram of the web‐based Lnc2m6A resource. (A) The query of m^6^A‐related lncRNAs in cancer. (B) The query of m^6^A‐related lncRNAs involved ceRNA networks in cancer. (C) The query of m^6^A‐related lncRNAs involved drug sensitivity in cancer. (D) The query of m^6^A‐related lncRNAs involved survival results in cancer. (E) All resources on this website can be downloaded for further analysis.

To help researchers apply the strategy, a comprehensive resource Lnc2m6A (http://hainmu‐biobigdata.com/Lnc2m6A) was developed to describe any cancer type of interest. This platform can query the lncRNAs or m^6^A regulators of interest and obtain their associations in a specific cancer type (Figure 6A). Lnc2m6A also provided three useful tools, which include (i) constructing the m^6^A‐mediated ceRNA networks in cancer (Figure 6B), (ii) exploring the relationship between m^6^A‐related lncRNA and drug resistance in a specific cancer context, based on ceRNA networks (Figure 6C), and (iii) investigating the clinical associations of m^6^A‐related lncRNAs (Figure 6D). All the generated data in this work can be downloaded for further analysis (Figure 6E). In conclusion, the comprehensive resource Lnc2m6A (which will be continuously updated) could be used to prioritize m^6^A‐related lncRNA molecules and further explore their essential functions in human tumors.

## 4 DISCUSSION

m^6^A modification widely exists in diverse noncoding RNAs and tightly regulates lncRNAs, thereby influencing tumorigenesis and cancer development.^44^, ^45^ This study comprehensively identified m^6^A‐related lncRNAs and explored their distribution across 33 cancer types. The results were verified in another large sample cohort. There was a broad spectrum of positive correlations between lncRNAs and m^6^A regulators across cancer types, whereas considerable negative correlations were only observed in TGCT and THYM. Moreover, these m^6^A‐related lncRNAs were with significantly different genomic features than other lncRNAs, which include CpG content, number of CpG, and RRACH motifs. In particular, cancer‐common and cancer‐specific m^6^A‐related lncRNAs that play important roles in cancer progression were defined. These two categories of lncRNAs also showed different characteristics in expression deviation, CpG content, conservation, and a number of related m^6^A regulators. This study found that cancer‐specific lncRNAs were associated with tissue specificity, also that cancer‐common lncRNAs were conserved in cancer‐related biological function.

Previous studies have revealed the critical roles of lncRNAs involved in various carcinogenic mechanisms in cancers.^46^, ^47^, ^48^ The numerous expressions of lncRNAs associated with m6A regulators were revealed in this study, such as LINC00920 (LINRIS), DLEU2 (LINC00022), LINC00958, and NEAT1. The results of this study are broadly in accordance with recent studies on m6A modification in various cancer types. For example, LINC00920 blocked the ubiquitination of IGF2BP2 and maintained the MYC‐mediated glycolysis process in colorectal cancer.^48^ The upregulation of DLEU2 promoted tumorigenesis of esophageal squamous cell carcinoma, which was epigenetically mediated by m6A demethylase FTO.^49^ Zou et al. found that METTL3‐mediated lncRNA LINC00958 increased lipogenesis and served as a nanotherapeutic target for liver cancer.^12^ The mutation on the m6A sites of NEAT1 inhibited the metastasis of prostate cancer cells.^50^ Collectively, these findings highlight the important roles of the m6A modification in regulating lncRNAs and verified the reliability of our methods.

Notably, several key m^6^A‐related lncRNAs which were novel discoveries or had been verified in literature were identified. Li et al. found that lncRNA FGD5‐AS1 was overexpressed in breast cancer tissues and predicted poorer clinical characteristics and prognosis.^51^ This study demonstrated that most m^6^A‐related lncRNA FGD5‐AS1 was associated with cisplatin resistance, by competitively binding to has‐miR‐362‐3p in BRCA patients. In another case, Hu et al. revealed that IGF2BP2 served as an m^6^A reader to regulate lncRNA DANCR, thereby promoting cancer stemness‐like properties and pathogenesis.^52^ Through bioinformatics analysis, DANCR was found to be significantly associated with m^6^A regulators in LGG (Figure S8A). Its ceRNA network in LGG comprised considerable glioma and central nervous system (CNS) development‐related genes (obtained from NCG v6.0^53^ and MSigDB v7.4^54^) (Figure S8B). Moreover, the functional enrichment and survival analysis revealed that DANCR was related to the mRNA metabolic process and patient OS in LGG (Figure S8C,D). All these results suggest the important roles of key m^6^A‐related lncRNAs in tumor pathogenesis and development.

This study conducted a user‐friendly webserver to query the m^6^A‐participated ceRNA network, survival molecules, and drug sensitivity which focused on lncRNA as the entry point. Significant associations between lncRNAs and m^6^A regulators have been identified and included in Lnc2m6A. Compared with other m^6^A‐related resources such as M6ADD^55^ and M6A2Target,^23^ Lnc2m6A is the first database that specifically focused on the lncRNAs which related to m^6^A writers, readers, and erasers. Users can obtain desired associations restricted to specific RNA molecules and cancer types. More importantly, Lnc2m6A integrated three user‐friendly tools, which explored the potential function of m^6^A‐related lncRNAs based on the principles described in this study. Through the construction of ceRNA networks, drug resistance estimation, and survival analysis, Lnc2m6A may help researchers to characterize and reveal the function of m^6^A‐related lncRNAs, in the context of human tumors. In addition, Lnc2m6A also has a limitation. This study only estimated the association between lncRNAs and m^6^A regulators based on the public cohort, which may cause the results to be slightly limited. The extensions of Lnc2m6A will continue, newly comprehensive and reliable versions of the database will solve these problems in the future. Further experimental and theoretical investigations could be directly performed based on the information on this platform.

## 5 CONCLUSION

In summary, this study comprehensively analyzed the landscape of m^6^A‐related lncRNAs across 33 human cancer types. Along with the exploration of the associations between lncRNAs and m^6^A regulators, this study also proposed a systematical strategy to reveal the potential functions of m^6^A‐related lncRNAs. Continued investigations on these essential lncRNAs will deepen the understanding of tumorigenesis and cancer treatment in the epigenetics field.

## AUTHORS' CONTRIBUTION

K.L., J.Y., and D.W. designed the study, D.X., Z.X. X.B., J.C., C.M., D.Z., L.C., and P.L. H.W. analyzed and interpreted the data, D.W., D.X., Z.X., X.B., and J.C. constructed the web‐based resource, D.X., K.L., and Z.X. wrote and edited the manuscript, and all the authors read and approved the manuscript.

## FUNDING INFORMATION

This work was supported by the Major Science and Technology Program of Hainan Province (ZDKJ202003, ZDKJ2021040), Hainan Provincial Natural Science Foundation of China (820RC637), the Key Research and Development Project Of Hainan Province (ZDYF2021SHFZ097), National Natural Science Foundation of China (32160152), Innovative research project for Graduate students in Hainan Province (Hyb2020‐56, Hys2020‐378), and Hainan Province Clinical Medical Center.

### ETHICS APPROVAL AND CONSENT TO PARTICIPATE

Not applicable.

### CONSENT FOR PUBLICATION

Not applicable.

### CONFLICT OF INTEREST

The authors declare that they have no competing interests.

## Supporting information


Figure S1
Figure S2
Figure S3
Figure S4
Figure S5
Figure S6
Figure S7
Figure S8

Table S1
Table S2
Table S3

## Data Availability

The transcriptome profiles and clinical data can be found at UCSC Xena (http://xena.ucsc.edu/). Software and resources used for the analyses are described in each method section.
