# Supplementary material for: Identification and functional analysis of N6‐methyladenine (m6A)‐related lncRNA across 33 cancer types
Source: Cancer Med. 2022 Jul 4;12(2):2104–16. doi: 10.1002/cam4.5001 (PMC9883401; doi:10.1002/cam4.5001)
Supplement: Supplementary file 1 — Figure S1 Figure S2 Figure S3 Figure S4 Figure S5 Figure S6 Figure S7 Figure S8 [file CAM4-12-2104-s002.docx]

**
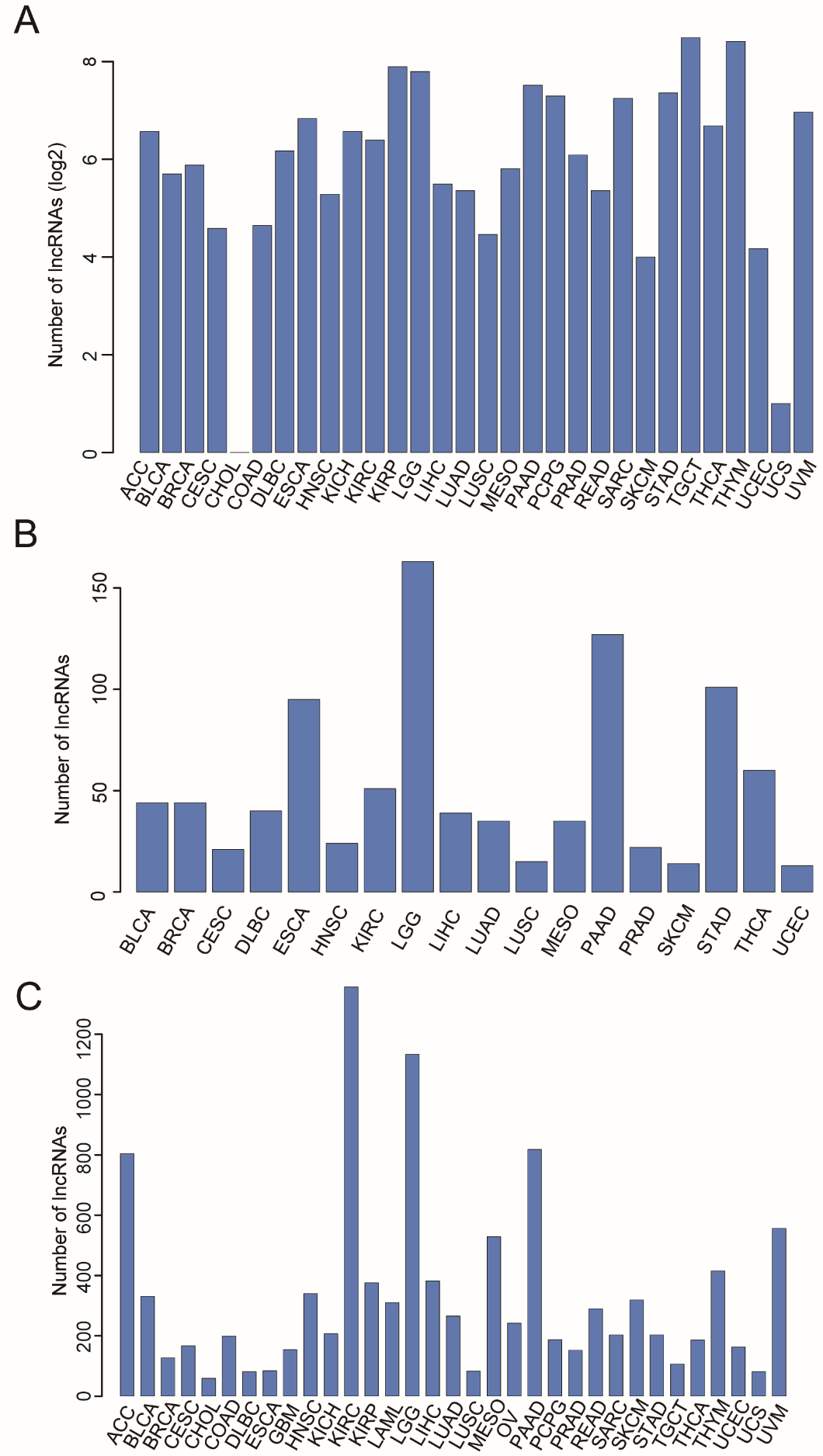
**

**Figure S1. Number of m^6^A-related lncRNA with putative biogenesis across cancer types.** Number of lncRNAs involved in (A) ceRNA networks (B) drug sensitivity and (C) clinical survival.

**
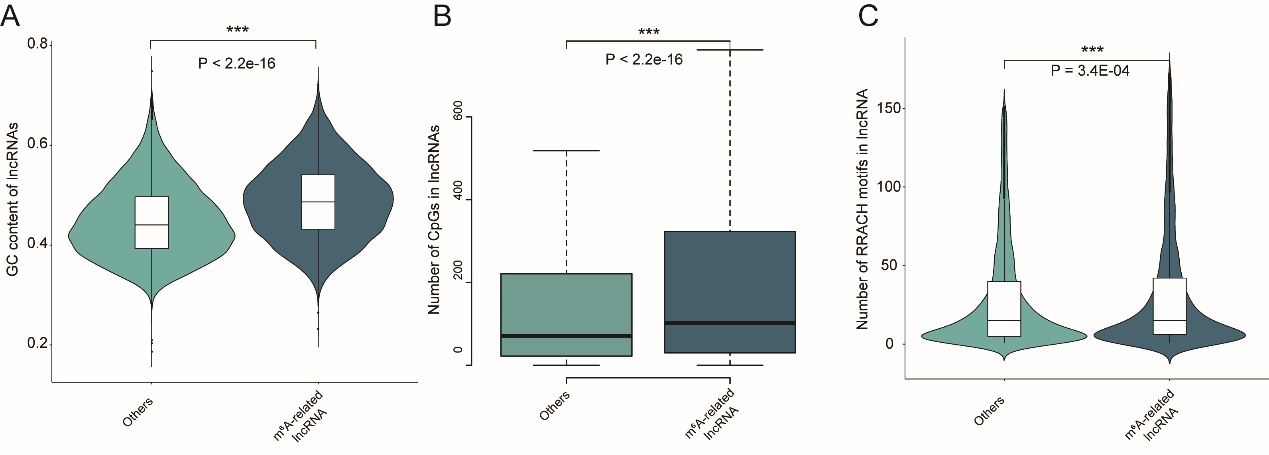
**

**Figure S2. Characterization of m6A-related and other lncRNAs.** (A) The comparision of GC content between m6A-related and other lncRNAs. (B) Box plots showing the number of CpG between m6A-related and other lncRNAs. (C)The number of RRACH motif between m6A-related and other lncRNAs.


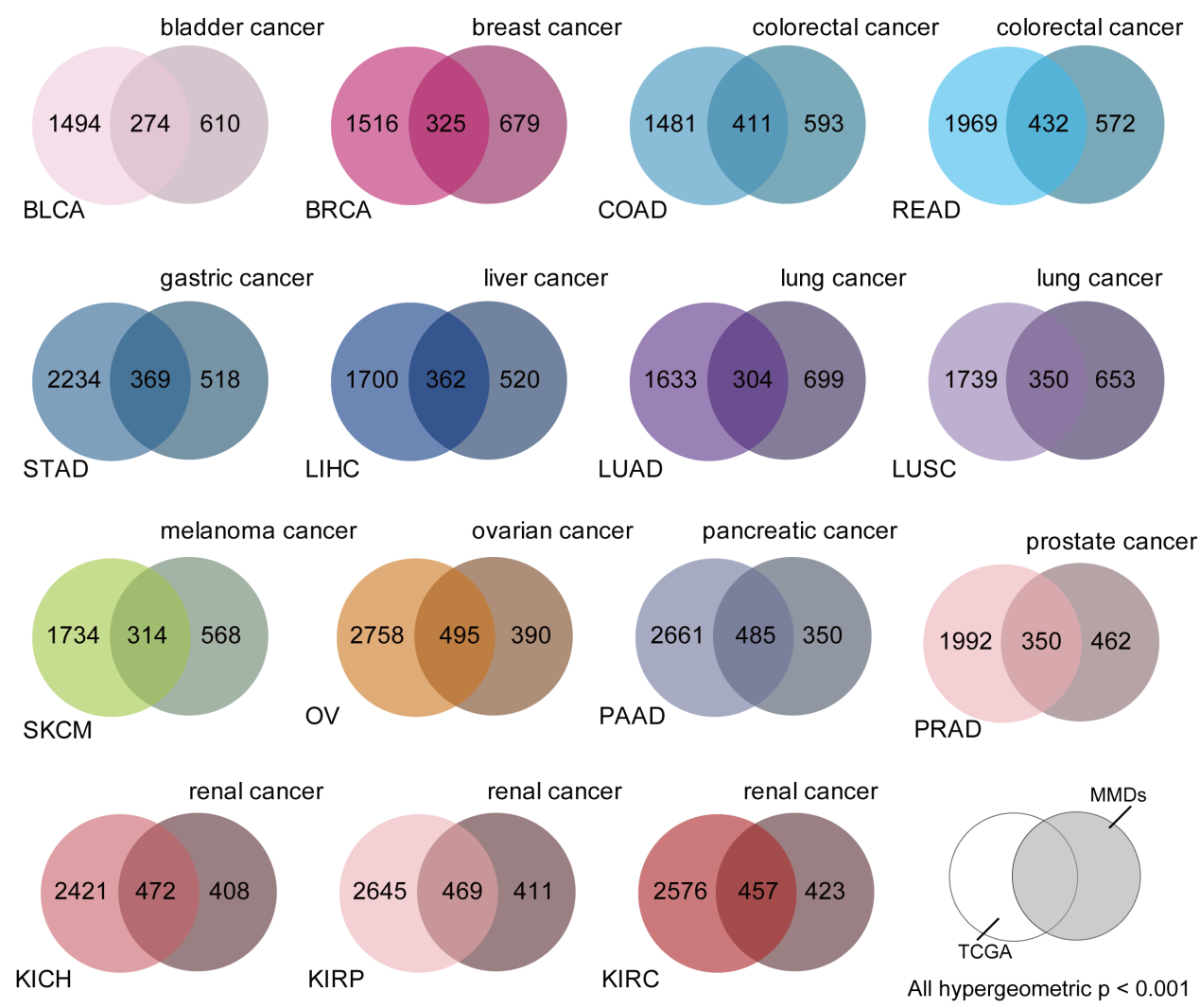


**Figure S3. Overlap of m^6^A-related lncRNAs between TCGA and MMDs datasets.**

**
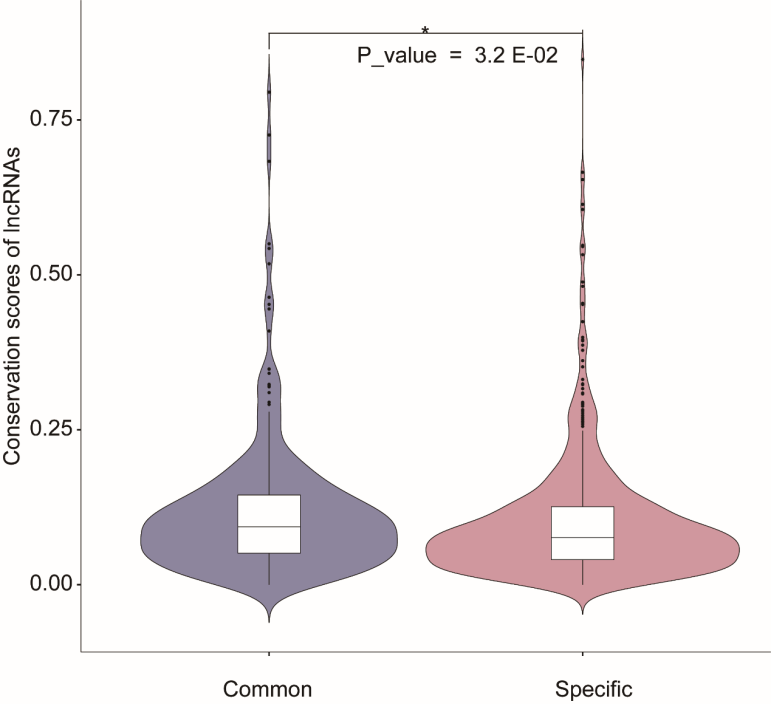
**

**Figure S4. The comparision of conservation scores between cancer-common and cancer-specific m^6^A-related lncRNA.**

**
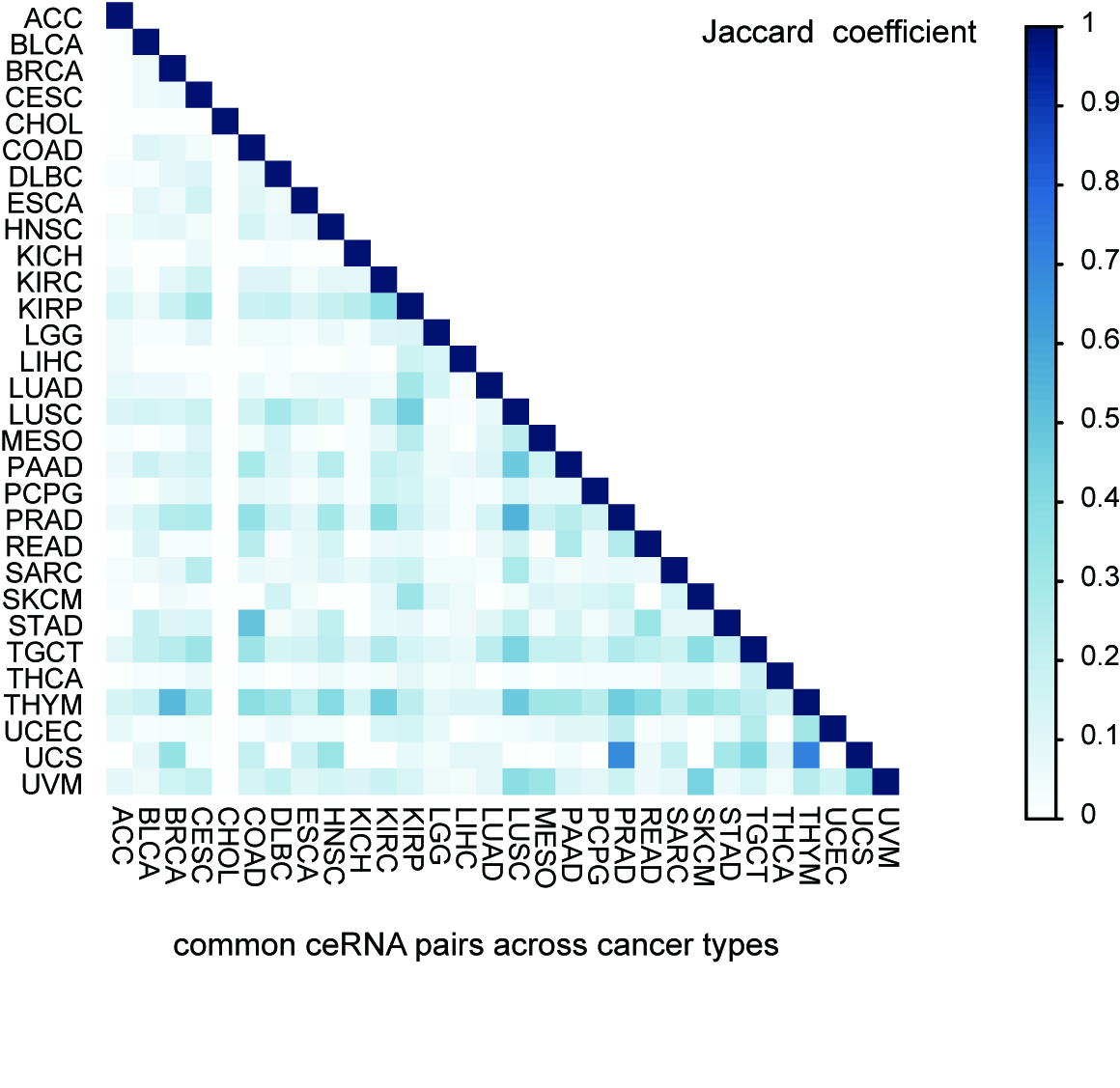
**

**Figure S5. The Simpson coefficient of ceRNA pairs across cancer types.**

**
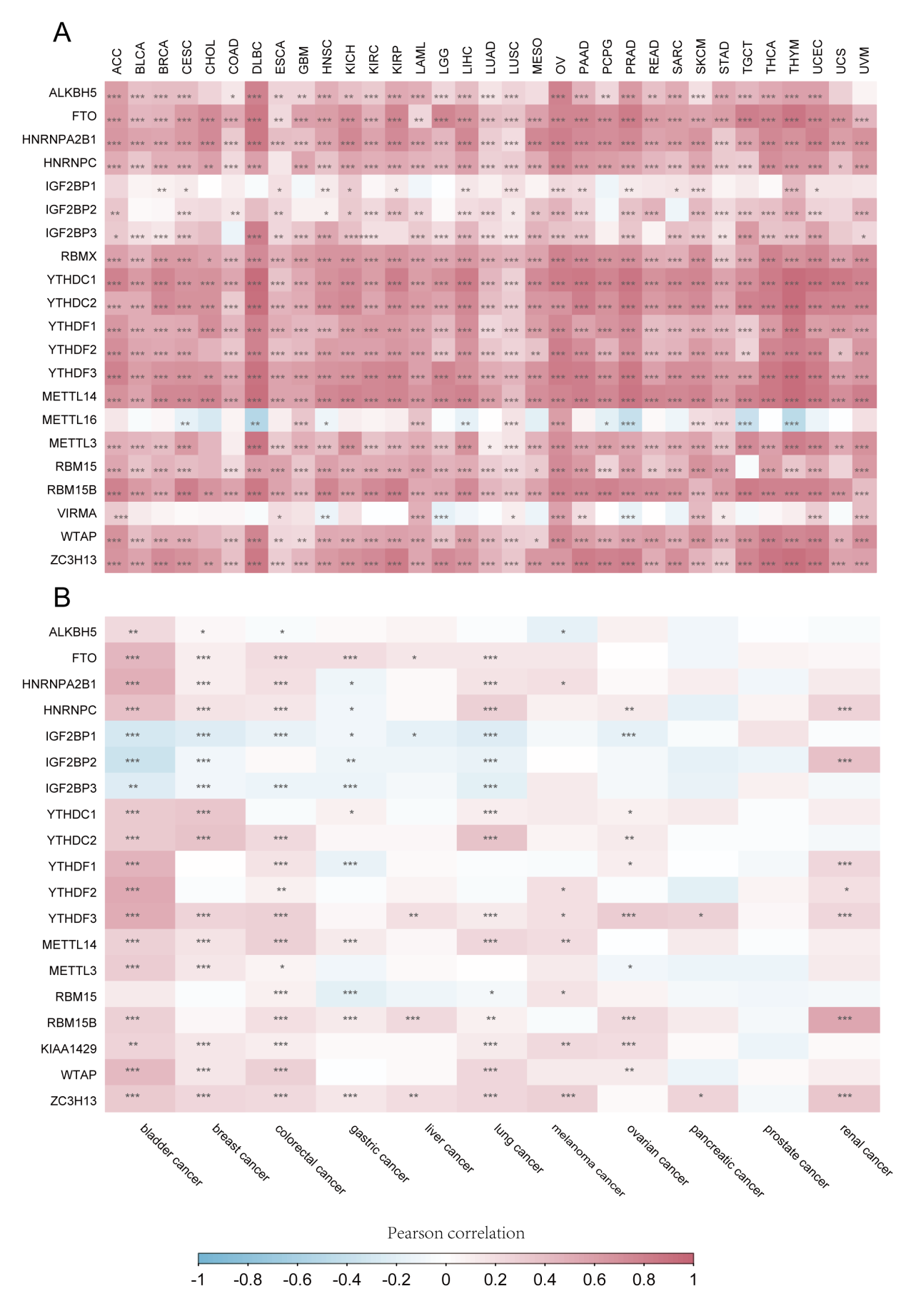
**

**Figure S6. Correlation between the expression level of FGD5-AS1 and m^6^A regulators in (A) TCGA and (B) MMDs datasets.**

**
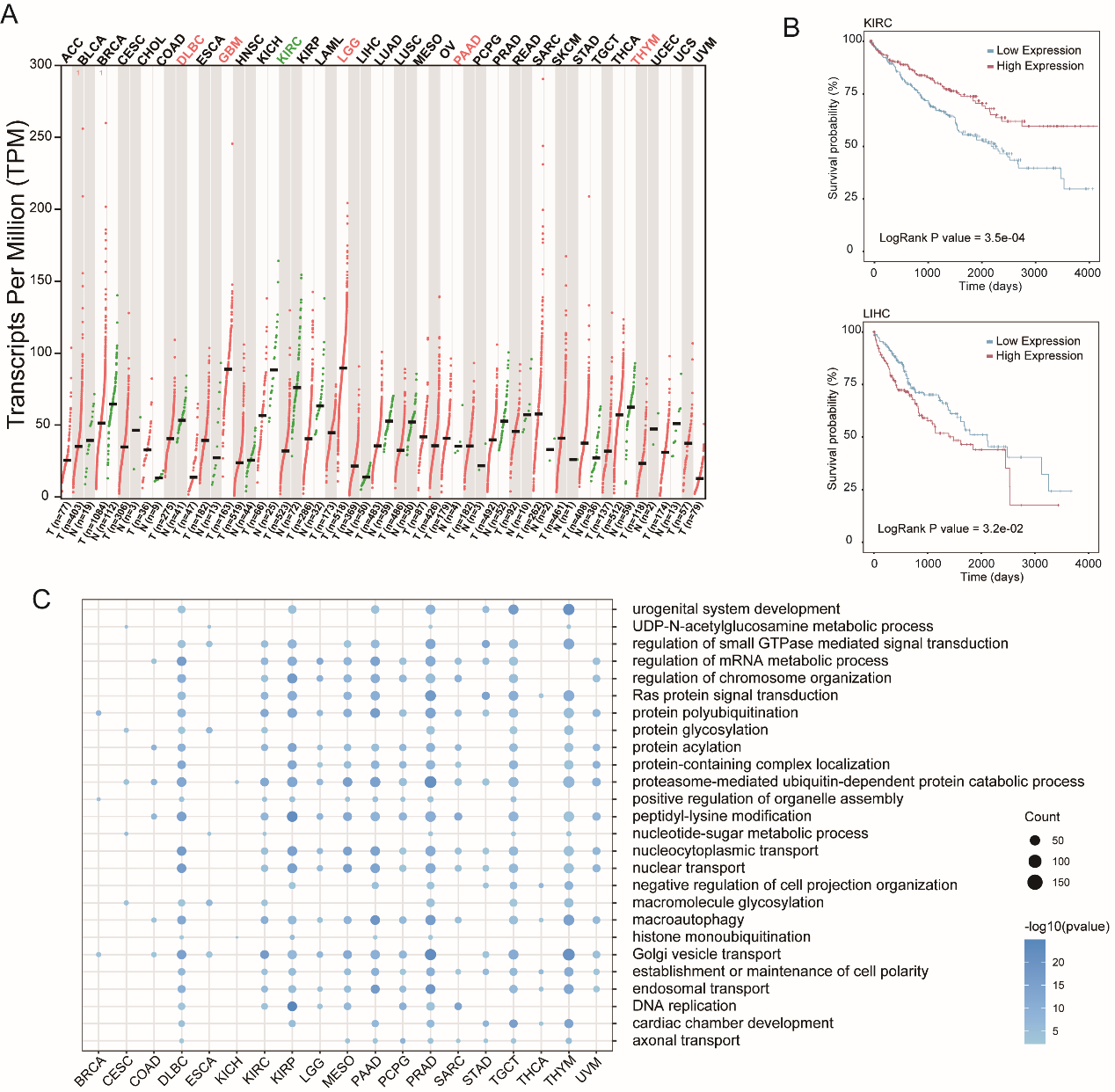
**

**Figure S7. Functional analysis of m^6^A-related lncRNA FGD5-AS1 in pan-cancer.** (A) The differential expression analysis of FGD5-AS1 in pan-cancer. (B) Survival analyses for patients with low and high FGD5-AS1 expression in the KIRC (top) and LIHC (bottom) cohorts. (C)The functional enrichment analysis of FGD5-AS1 in pan-cancer.

**
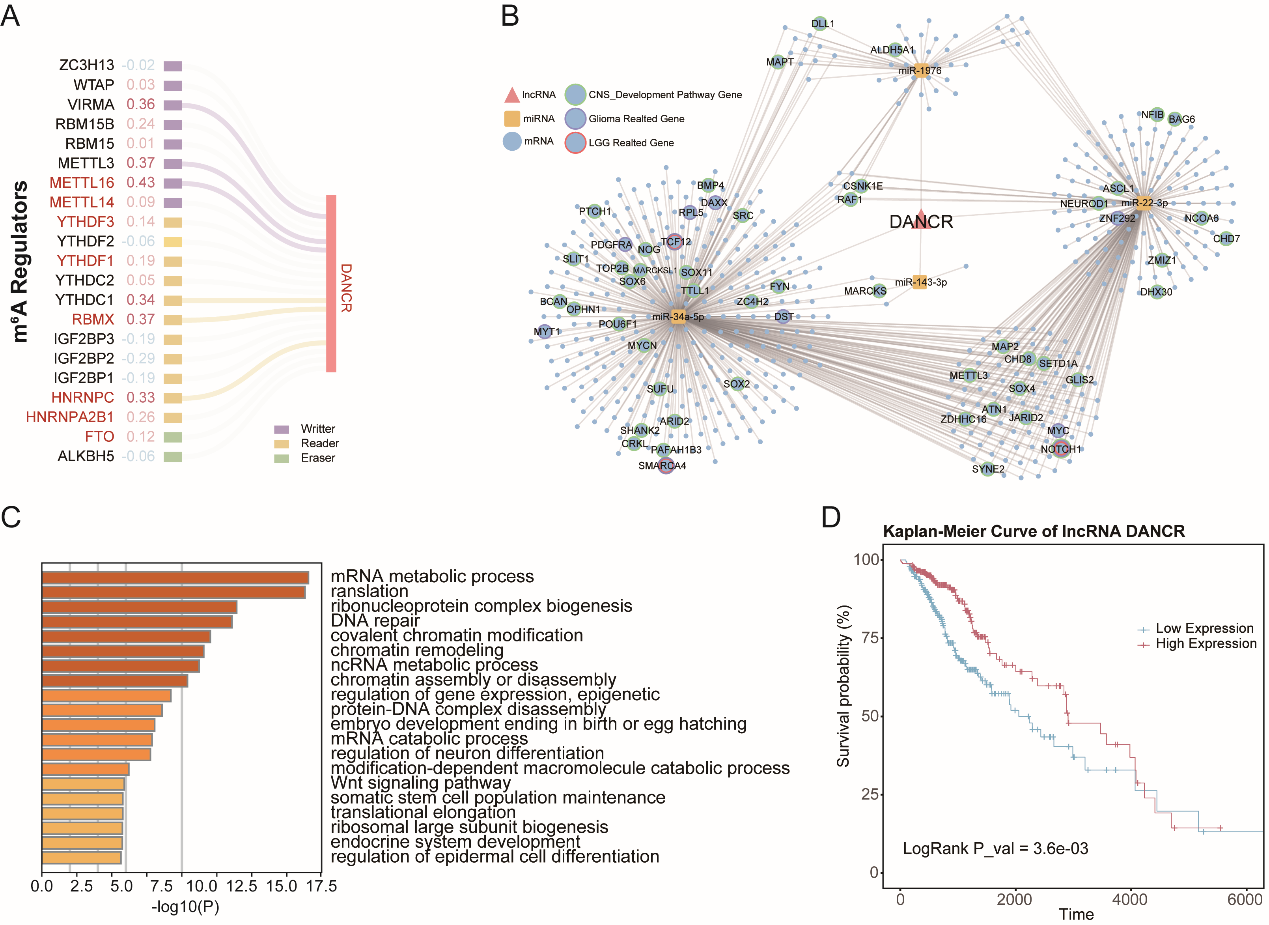
**

**Figure S8. m^6^A-related lncRNA DANCR plays essential roles in LGG.** (A)The correlation between DANCR and 21 m6A regulators. (B) The ceRNA network of DANCR in LGG. (C) Functional enrichment analysis of DANCR based on LGG ceRNA network. (D)Survival analyses for patients with low and high DANCR expression in LGG.
